# Long Read Single-Molecule Real-Time Sequencing Elucidates Transcriptome-Wide Heterogeneity and Complexity in Esophageal Squamous Cells

**DOI:** 10.3389/fgene.2019.00915

**Published:** 2019-10-04

**Authors:** Yin-Wei Cheng, Yun-Mei Chen, Qian-Qian Zhao, Xing Zhao, Ya-Ru Wu, Dan-Ze Chen, Lian-Di Liao, Yang Chen, Qian Yang, Li-Yan Xu, En-Min Li, Jian-Zhen Xu

**Affiliations:** ^1^The Key Laboratory of Molecular Biology for High Cancer Incidence Coastal Chaoshan Area, Shantou University Medical College, Shantou, China; ^2^Department of Biochemistry and Molecular Biology, Shantou University Medical College, Shantou, China; ^3^Computational Systems Biology Lab, Department of Bioinformatics, Shantou University Medical College (SUMC), Shantou, China; ^4^Tianjin Novogene Bioinformatics Technology Co., Ltd, Tianjin, China; ^5^China Institute of Oncologic Pathology, Shantou University Medical College, Shantou, China

**Keywords:** heterogeneity, long reads sequencing, esophageal squamous cell carcinoma, transcriptome, alternative splicing, lincRNA, transcript fusion

## Abstract

Esophageal squamous cell carcinoma is a leading cause of cancer death. Mapping the transcriptional landscapes such as isoforms, fusion transcripts, as well as long noncoding RNAs have played a central role to understand the regulating mechanism during malignant processes. However, canonical methods such as short-read RNA-seq are difficult to define the entire polyadenylated RNA molecules. Here, we combined single-molecule real-time sequencing with RNA-seq to generate high-quality long reads and to survey the transcriptional program in esophageal squamous cells. Compared with the recent annotations of human transcriptome (Ensembl 38 release 91), single-molecule real-time data identified many unannotated transcripts, novel isoforms of known genes and an expanding repository of long intergenic noncoding RNAs (lincRNAs). By integrating with annotation of lincRNA catalog, 1,521 esophageal-cancer-specific lincRNAs were defined from single-molecule real-time reads. Kyoto Encyclopedia of Genes and Genomes enrichment analysis indicated that these lincRNAs and their target genes are involved in a variety of cancer signaling pathways. Isoform usage analysis revealed the shifted alternative splicing patterns, which can be recaptured from clinical samples or supported by previous studies. Utilizing vigorous searching criteria, we also detected multiple transcript fusions, which are not documented in current gene fusion database or readily identified from RNA-seq reads. Two novel fusion transcripts were verified based on real-time PCR and Sanger sequencing. Overall, our long-read single-molecule sequencing largely expands current understanding of full-length transcriptome in esophageal cells and provides novel insights on the transcriptional diversity during oncogenic transformation.

## Introduction

Esophageal squamous cell cancer (ESCC) is a serious malignancy with poor prognosis and mortality rate (Lin et al., 2013; Chen et al., 2016). Recently, large-scale sequencing studies have revealed the substantial genomic heterogeneity within and among ESCC patients, which hampered the development of effective target therapies (Hao et al., 2016; Yan et al., 2019). While genetic alterations initiate tumorigenesis, how they affect the transcriptional program and ultimately drive the malignant phenotype remains elusive. In order to find the altered signaling pathways and novel functional transcripts such as long intergenic noncoding RNAs (lincRNAs), several short-read based transcriptome sequencing studies have been conducted during the past several years (Cancer Genome Atlas Research et al., 2017; Li et al., 2017). However, typical RNA-seq captures a large number of contiguous short reads (about 100–250 bp) and reconstructs the transcripts by statistical modeling. Thus, it is difficult to completely describe RNA molecules from 5¢ to 3¢ end and to annotate novel isoforms or genes using short reads (Au et al., 2013; Steijger et al., 2013). On the contrary, PacBio single-molecule real-time (SMRT) platform is capable to sequence long circular-consensus sequence reads of several thousand base pairs and have a good opportunity to capture full-length transcripts de novo. In addition, hybrid sequencing algorithms have been developed to correct sequencing errors by utilizing high accurate short reads. Thus, hybrid PacBio SMRT sequencing provides a powerful tool to survey the transcriptional landscape in cells (Sharon et al., 2013; Tilgner et al., 2014; Weirather et al., 2015).

In this study, we selected one normal immortalized esophageal squamous epithelial cell line and four ESCC cell lines, which represent major cell types of esophageal squamous cell carcinoma (ESCC) and investigated the cellular heterogeneity at transcriptome level. Using hybrid PacBio SMRT platform, our de novo sequencing of these five representative esophageal cell lines yields ∼210 Gb of clean data or ∼2,000,000 full-length nonchimeric (FLNC) reads. All of these FLNC reads have clear 5¢ and 3¢ messenger RNA (mRNA) canonical structure and with an average length of >2.5 kb, which are well suitable to describe the full transcript structures. We detected many new transcripts such as novel isoforms, esophageal cancer specific lincRNAs and gene fusions; we also cataloged the shifted alternative splicing (AS) feature between cancer and normal esophageal cells, which collectively highlighting the true heterogeneity and complexity.

## Methods

### Sample Preparation

Four human ESCC cell lines [KYSE140, KYSE510, TE5, and Shantou human embryonic esophageal carcinoma (SHEEC)] and one normal immortalized esophageal squamous epithelial cell line (SHEE) were used in this study. Among the five cell lines in this study, KYSE140, KYSE510, and TE5 are established from the resected specimens of patients with ESCC (Shimada et al., 1992; Nishihira et al., 1993). We chose these three ESCC patient-derived cell lines as they cover all three types of cell differentiation of primary tumor: KYSE140 is derived from a patient with moderately differentiated squamous cell carcinoma, KYSE510 is derived from a patient with well-differentiated squamous cell carcinoma, and TE5 is derived from a patient with poorly differentiated squamous cell carcinoma. The SHEE and SHEEC cell lines were previously established by our labs. SHEE cell line was a HPV18 E6E7-immoltalized human embryonic esophageal epithelial cell line (Shen et al., 2001), and SHEEC cell line is tumor cell line established by malignant transformation of SHEE induced by 12-O-tetradeeanoyl-phorbol-13-acetate (TPA) (Shen et al., 2000). The cell lines were authenticated by short-tandem repeat analysis in 2018. Briefly, KYSE140 and KYSE510 cells were cultured in RPMI 1640 medium (Thermo) containing 10% fetal bovine serum (GIBCO). TE5 cell was cultured in Dulbecco’s modified Eagle’s medium (Thermo Fisher Scientific) supplemented with 10% fetal bovine serum. SHEEC and SHEE cells were cultured in Dulbecco’s modified Eagle’s medium/F12 medium (Thermo Fisher Scientific) with 10% newborn bovine serum (Excell Biology). All cell lines were tested without mycoplasma contamination before RNA isolation. Total RNA was isolated with TRIzol (Invitrogen) as per the manufacturer’s instructions and then treated with DNase and purified with PureLink^®^RNA Mini Kit (Life Technology). RNA was purified according to the following criteria: (1) with concentration ≥300 ng/µl, (2) OD260/280 = 2.0–2.2 and OD260/230 = 1.8–2.1, and (3) RNA integrity number (RIN) ≥9, which is assessed on the Agilent Bioanalyzer 2100 system.

### Library Preparation and Sequencing

The SMRTbell^™^ libraries were prepared according to the Isoform Sequencing protocol (Iso-Seq) as described by Pacific Biosciences (PN100-092-800-03). First, the complementary DNA (cDNA) was synthesized by total mRNAs for each sample using the Clontech SMARTer PCR cDNA Synthesis Kit. To increase the sequencing yield of long transcripts, the Blue Pippin Size Selection System protocol was used to select the >4-kb fragments for each sample after the PCR amplification of the cDNAs. Then, the equimolar mixture of long cDNA fragments (>4 kb) and normal cDNAs was used subsequently for SMRT sequencing. For Illumina transcriptome library preparation and sequencing, a total amount of 3 µg RNA per sample was used as input material for the RNA sample preparations. Sequencing libraries were generated using NEBNext^®^ UltraTM RNA Library Prep Kit for Illumina^®^ (NEB, USA) following manufacturer’s recommendations. The Illumina PE150 libraries were sequenced on Hiseq 4000 platform.

### Raw Data Processing and Mapping to Reference Genome

SMRT data were processed using the SMRTlink 5.0 software (Pacific Biosciences). Circular consensus sequence was generated from subread BAM files with the default parameters. The nonchimeric reads, which include nonfull-length and full-length transcripts, were then clustered by isoform level clustering algorithm. The produced clusters were finally polished using ARROW software (Pacific Biosciences). Additional nucleotide errors in consensus reads were corrected using the Illumina RNA-seq data by the LoRDEC software (Salmela and Rivals, 2014). Consensus reads were aligned to reference annotations (Ensemble 38 release 91) using GMAP (Wu and Watanabe, 2005) with the following parameters –no-chimeras –cross-species –expand-offsets 1 -B 5 -K 50000 -f samse -n 1.

For Illumina RNA-seq reads mapping, reference genome and gene model annotation files were downloaded from genome website directly (ftp://ftp.ensembl.org/pub/release-91/fasta/homo_sapiens/dna/Homo_sapiens.GRCh38.dna.primary_assembly.fa.gz). Hisat2, which is a fast-spliced aligner with low-memory requirements, was used to build the index of the reference genome, and paired-end clean reads were mapped against the reference genome (https://github.com/infphilo/hisat2). HTSeq v0.6.1 was used to count the read numbers mapped to each gene. Then, read per kilobase of exon per million mapped reads of each gene was calculated based on the length of the gene and reads count mapped to this gene (http://htseq.readthedocs.io/en/release_0.9.1/).

### Gene Structure Analysis and Novel Transcript Annotations

The GMAP output BAM format file and GTF format genome annotation file were used for gene and transcript structure determination. Long read clusters were overlapped with gene models to find novel isoforms and genes as previously reported (Abdel-Ghany et al., 2016). We also compared the transcription start sites (TSS) of each transcript with the Cap Analysis of Gene Expression (CAGE) promoter tags and epigenetic marks that are typically associated with actively transcribed promoters (H3K4me1, H3K4me3, and H3K27ac). TSSs are defined as the first genomic position of each transcript structure. We downloaded peak calls of CAGE promoter tags from FANTOM5 (FANTOM Consortium and the RIKEN PMI and CLST (DGT) et al., 2014) and epigenetic marks (adult esophagus) from the Roadmap Epigenomics Consortium (Bernstein et al., 2010). *LiftOver* was used when necessary (https://genome.ucsc.edu/cgi-bin/hgLiftOver). 

Unmapped transcripts and novel gene transcripts were scanned and annotated by Diamond BLASTX with parameter e value “1e−5” in the following protein/peptide databases (Buchfink et al., 2015): NR (NCBI nonredundant protein sequences), KOG/COG (Clusters of Orthologous Groups of proteins), Swiss-Prot, Kyoto Encyclopedia of Genes and Genomes (KEGG) Ortholog database. Similarly, novel transcripts were also searched against the Pfam database (Finn et al., 2016) by Hmmscan software (http://hmmer.org/download.html).

### Analysis of Alternative Splicing Patterns

Alternative events were analyzed by SUPPA (Trincado et al., 2018). To quantify the differential isoform usage between cells, we defined the score D of each gene as follows:

(1) $$D_{j}=\sum_{i=1}^{4}\left(1-\frac{c_{i}}{d}\right)\text{ whereas }c=a\cap b,\;d=a\cup b$$

suppose gene *j* has isoform set a and set *b*, respectively, in cell line X and Y; *c* is the number of isoform intersection for set *a* and set *b*; *d* is the number of isoform union for set *a* and set *b*. Thus, *D* sums up scores when comparing the four esophageal squamous carcinoma cells with SHEE. Genes with a higher *D* value are more diversely spliced. A set of differentially spliced genes, which was identified from TCGA clinical esophageal samples, were used in this study to verify the altered splicing pattern (Mao et al., 2019). Enrichment analysis of spliced genes was conducted by DAVID against GO Biological Processes terms with a cutoff false discovery rate ≤ 0.05 (Huang da et al., 2009).

### lncRNA Analysis Pipeline

SMRT transcripts were first analyzed by CNCI and PLEK with default parameters to predict the coding potential of transcripts. These two software adopted support vector machine algorithms to effectively distinguish protein-coding and noncoding sequences independent of known protein annotations (Sun et al., 2013, Li et al., 2014a). In order to strictly identify the lncRNA candidates, SMRT transcripts were also scanned against known protein sequence databases subsequently. CPC software was used to assess the extent and quality of the open reading frame in a transcript and search the sequences against NCBI eukaryotes’ protein database to clarify the coding and noncoding transcripts (Kong et al., 2007). Pfam-scan translated each transcript in all three possible frames to identify occurrence of any of the known protein family domains documented in the Pfam database (Finn et al., 2016). Finally, transcripts predicted with coding potential by any of the above four tools were filtered out, and those without protein coding potential were candidate set of lncRNAs. The Cabili’s reference set, which catalogs lincRNAs across 24 human tissues and cell types (but without esophageal tissue), were downloaded from supplemental files of the publication (Cabili et al., 2011). Candidate lincRNAs from four esophageal cancer cells, but not expressed in SHEE cells and Cabili’s reference set, were defined as esophageal cancer cell specific lincRNAs.

The interacting target genes of lncRNAs are predicted based on an approach using the coexpression and colocalization pattern (Dempsey et al., 2018, Gao et al., 2019). Briefly, the expression correlation was calculated between lncRNAs and coding genes. Genes with a Pearson correlation coefficient >0.95 (*p* < 0.001) and reside in 100k upstream or downstream of lncRNA were identified as target genes of that lncRNA.

All microRNA (miRNA) hairpin sequences were downloaded from miRBase database (http://www.mirbase.org) and were blasted against the sequences of esophageal cancer cell specific lincRNAs to identify potential pre-microRNA.

### Transcripts Fusion Detection and Comparison With Known Gene Fusion in Database or Predicted From RNA-Seq

SMRT transcripts were determined as transcript fusions according to the following criteria:(1) SMRT transcripts were mapped to two or more long-distance range separate loci, and each locus must map at least 10% of the query transcript; (2) total combined alignment coverage is at least 99%; (3) minimum distance between each mapped locus is above 100 kb; and (4) at least two Illumina reads were found spanning the junction to support the candidate fusion transcripts. SMRT transcripts, which meet all of above criteria, were regarded as fusion transcripts. The overviews of fusion events between locations in chromosomes are drawn in R package “RCircos” (Zhang et al., 2013).

Illumina RNA-seq reads were also used to detect gene fusion events by STAR-Fusion with default parameter (https://github.com/STAR-Fusion). Furthermore, based on comparing the number of Illumina RNA-seq reads spanning the junction (denoted as s) and reads adjacent to it on both sides (denoted as *a* and *b*, respectively), a set of candidate fusion transcripts with $\frac{a}{s}<2\cap \frac{b}{s}<2$ were filtered, then manually inspected for follow-up experimental verification. For comparison of detected fusions with known records in gene fusion database or predictions from RNA-seq data, we first transform the gene symbols to Ensemble Ids (which are used in ChimerDB 3.0); then, gene level match is considered as an overlap result.

### KEGG Pathway Enrichment Analysis

The KOBAS software was used for statistical enrichment of gene list based on KEGG pathways (Wu et al., 2006).

### Fusion Transcripts Verification and Sequencing

cDNA was prepared by SuperScript III (Invitrogen). Primers were designed to span the junctions and are listed in **Table S6**. Quantitative real-time PCR were conducted as previously reported (Xu et al., 2011). The expected PCR products for individual fusion were determined *via* melting curve analysis. PCR products were run on gels and then gel purified and subjected to Sanger sequencing (Shanghai Sangon Biotech Co., Ltd.).

Four RNA-seq projects (PRJNA140847 with 6 ESCC tumor tissue samples, PRJNA298963 with 15 tumor tissue samples, PRJNA435587 with 7 tumor tissue samples, and PRJNA147913 with 7 tumor tissue samples) were collected. Blast with default parameters was used to align fusion sequences to all of RNA-seq reads.

## Results

### Full-Length Transcriptome Sequencing of Esophageal Cells

**Figure 1:**
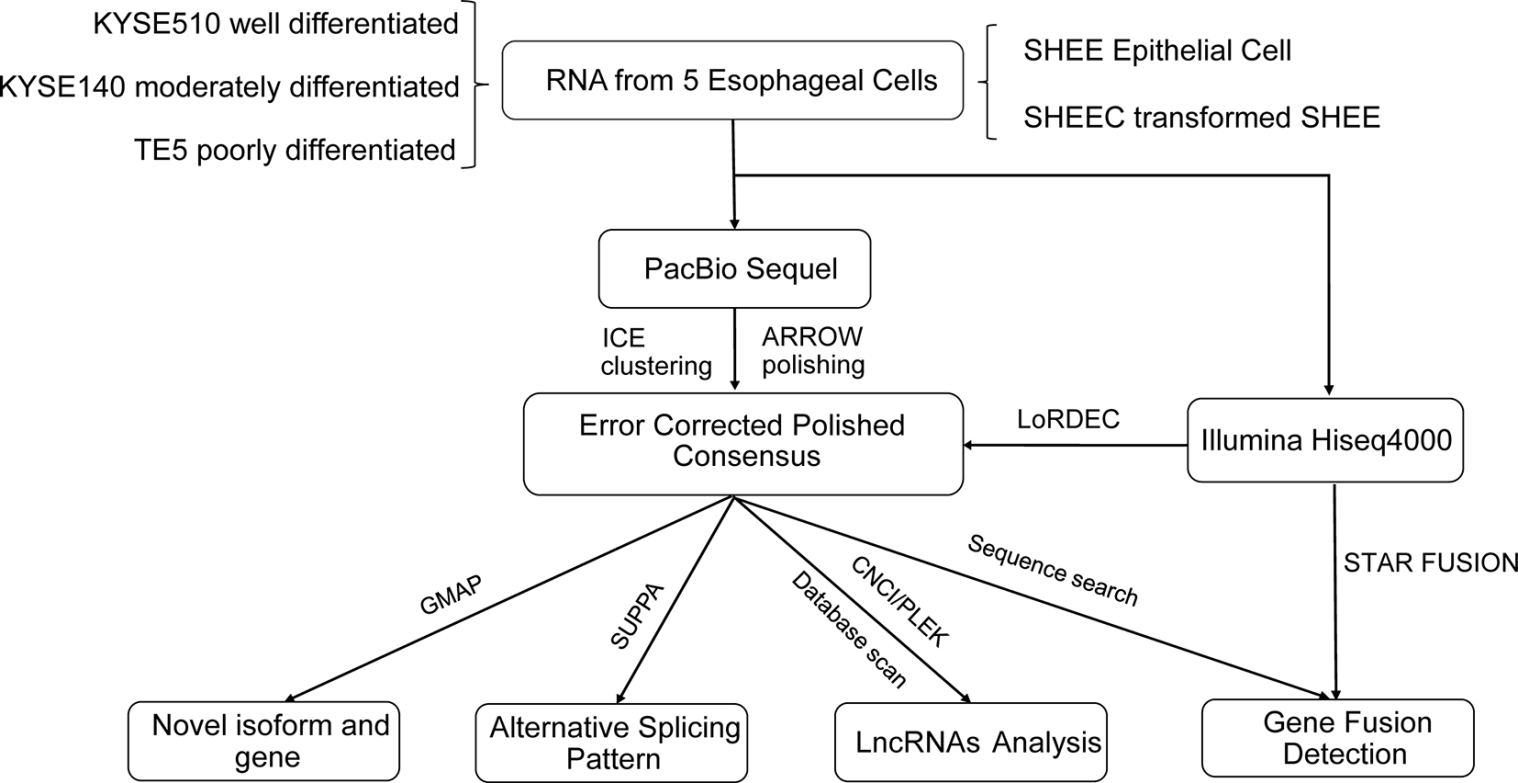
The analyses pipeline for transcriptional landscapes in esophageal cells.

The analysis pipeline for transcriptional landscape of esophageal cells is illustrated in **Figure 1**. According to Pacbio protocols, total RNA was purified to prepare for cDNA library for each cell line. Transcripts were simultaneously sequenced with SMRT cells on the PacBio Sequel and on the Illuminia Hiseq 4000 platforms. Totally, we identified 445,983, 477,033, 491,354, 327,459, 259,482 FLNC reads from KYSE140, KYSE510, SHEE, SHEEC, and TE5 cells, respectively, which cover ∼80% of all circular-consensus sequences in each cell line (**Table 1**). These high-quality FLNC reads were clustered by Arrow algorithm to obtain the consensus sequences. Short Illumina RNA-seq reads from the same RNA samples were also produced for each cell line. After trimming the sequencing adapter, poly(A) tail, and low-quality bases, the filtered RNA-seq reads were utilized to further correct FLNC reads by LoRDEC using the default parameters. After error correcting, the mean length of reads was within 2.3–3.2 kbp in esophageal cells, indicating good quality of the SMRT dataset (**Table S1A**).

**Table 1:** Quality control of single-molecule real-time (SMRT) raw data in esophageal cells.

Sample	CCS	Reads with 5′ primer	Reads with 3′ primer	Reads with Poly-A	Flnc	Average flnc read length	Flnc/CCS
KYSE140	557777	501,590	516,189	504,004	445,983	2,539	0.8
KYSE510	602754	545,561	558,823	539,496	477,033	2,442	0.79
SHEE	620818	555,241	574,085	559,246	491,354	2,566	0.79
SHEEC	424278	376,723	392,202	386,314	327,459	2,228	0.77
TE5	308010	284,929	287,900	283,066	259,482	3,073	0.84

CCS, Circular Consensus Sequence; Flnc, Full-length nonchimeric reads.

### Characterization of Full-Length Transcripts in Esophageal Cells

#### Novel Gene and Isoforms Identified From Full-Length Transcripts

**Figure 2:**
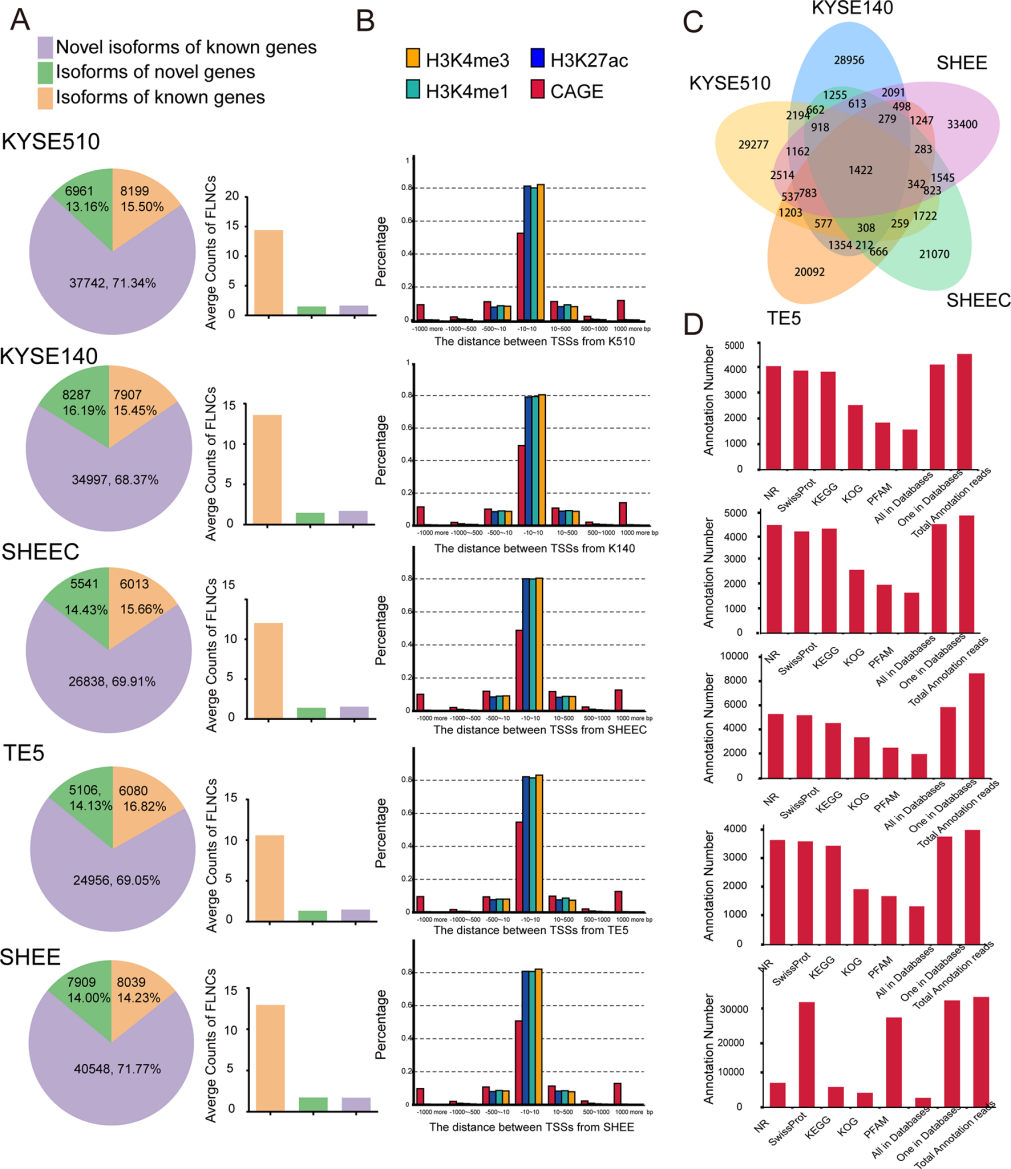
Single-molecule real-time (SMRT) sequencing identifies novel genes or isoforms of known genes. **(A)** Left panel: bar chart illustrates the percentages of novel isoform of known genes (purple), isoform of novel genes (green), and novel isoform of novel genes (brown). Right panel: average counts of full-length nonchimerics (FLNCs) in each esophageal cell line. **(B)** Distances distribution of transcription start site (TSS) in each full-length transcript to the closest epigenetic marks and Cap Analysis of Gene Expression (CAGE) tags. **(C)** The numbers of novel transcripts sharing among different cell lines. **(D)** Unannotated transcripts were scanned in several peptide or protein databases for each esophageal cell line.

We aligned long sequencing reads to the human Ensembl 38 release 91 genome using GMAP. We found an average alignment identity of ∼90%, with ∼80% transcripts uniquely mapping to the reference genome in each esophageal squamous cell (**Table S1B**). Similarly, short Illumina RNA-seq reads were also mapped to reference genome with a mapping rate >92% by HISAT2 mapper (**Table S1C**). According to the recent annotations, over 80% of SMRT transcripts are novel genes or isoforms of known genes (**Figure 2A** left panel). Compared with known isoforms of known genes, the expression of the novel isoforms from known or novel genes are relatively low (**Figure 2A** right panel). The numbers of novel transcripts sharing among different cell lines are demonstrated in **Figure 2C**.

To support the accuracy of obtained full-length transcripts and the analysis pipeline, we performed a transcriptome-wide comparison of TSSs detected in PacBio dataset with CAGE promoters and active epigenetic marks from Roadmap Epigenomics Project. Although these external datasets are generated from adult esophagus tissue, they are reasonable approximation for esophageal cells. As shown in **Figure 2B**, ∼50% of TSSs detected by long read sequencing were within 10 bp to their counterpart in the FANTOM5 CAGE dataset. They are even closer to the three epigenomics marks, and the majority of TSSs have a mean distance of 1 bp. The concordance between CAGE tags and epigenetic marks datasets with 5¢ ends detected in PacBio dataset confirmed the validity of identified full-length transcripts.

To further establish the accuracy of the full-length transcripts, those unannotated transcripts were blasted against several peptide or protein databases including NR, Pfam, KOG, KEGG, and Swiss-Prot. Protein products from over 85% of the transcripts can be found in at least one of the above databases, suggesting that many of the novel transcripts are indeed translated into proteins (**Figure 2D**). Overall, multiple orthogonal datasets provide independent confirmations that reported transcripts are most likely full length.

**Figure 3:**
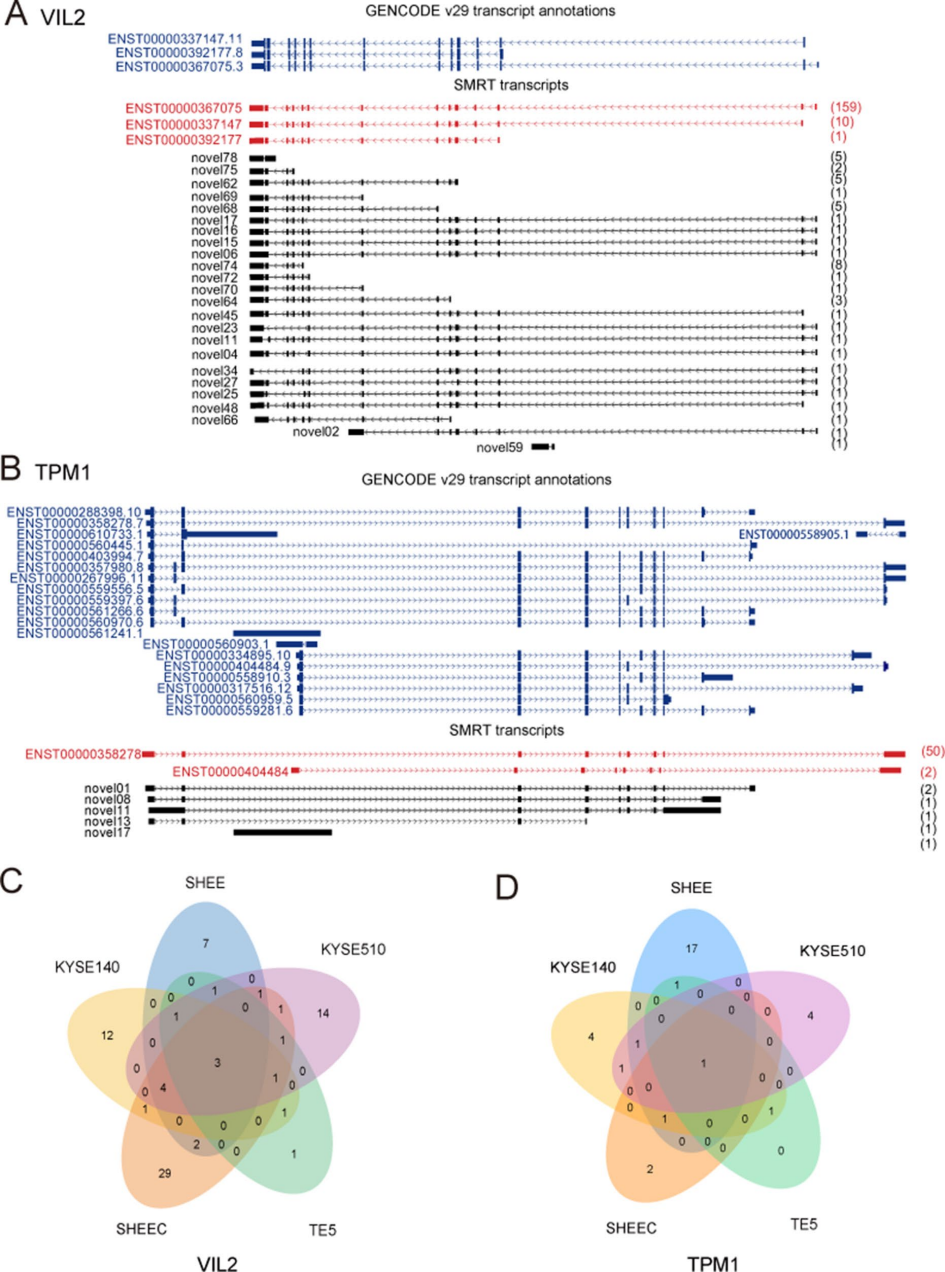
Examples of isoforms of known genes. Single-molecule real-time (SMRT) transcripts detected in each cell line for **(A)** VIL2 and **(B)** TPM1. **(C)** Novel isoforms of VIL2 gene in KYSE510 cells. **(D)** Novel isoforms of TPM1 gene in KYSE510 cells. Blue: Known transcript annotations; red: known isoforms identified from SMRT data; black: novel isoforms from SMRT data. The number of FLNCs detected is shown in brackets.

**Figure 3** provides two examples of these novel isoforms of known genes. For example, VIL2 (also known as Ezrin) encoded three transcript variants that differ in the transcriptional start site. We previously found that there are two variants of VIL2 in the esophageal cancer cells (V1: ENST00000337147.11;V2:ENST00000367075.3), and different transcriptional regulatory mechanisms regulated their transcription (Zhang et al., 2015; Zhang et al., 2018). From current SMRT data, it is clear that, except for the three annotated VIL2 variants in reference genome, other 24 VIL2 variants with different expressions are also transcribed but have not been annotated in KYSE510 cells (**Figures 3A, C**). We recently found that the AS of Tropomyosin I (TPM1) is regulated by its natural antisense TPM1-AS, resulting in specifically downregulation of TPM1variants (Huang et al., 2017). Similarly, multiple novel isoforms of this gene were also detected from SMRT data (**Figures 3B, D**). For both VIL2 and TPM1 genes, there are plenty of cell-type-specific variants than those shared by multiple cells, suggesting that there may be specific splicing events in each cell (**Figures 3C, D**).

To investigate what kind of genes have more novel isoforms, we sorted all genes according to the number of its isoforms and conducted the KEGG pathway enrichment analysis of top 10% genes with the most isoforms. We found that these genes are significantly enriched in known cancer-associated pathways such as viral carcinogenesis, PI3K-Akt, and MAPK signaling pathway (**Figure S1**).

#### Mining Esophageal Cancer Cell Specific lincRNAs

LincRNAs are transcribed from intergenic regions between protein-coding genes. Recent studies have pointed out their critical function associated with the pathogenesis of ESCC (Li et al., 2014b; Shen et al., 2016). Based on collections of ∼4 billion RNA-seq reads, Cabili et al. previously have defined a reference catalog of ∼14,000 human lincRNAs with expression pattern across 24 human tissues and cell types (without esophageal tissue) (Cabili et al., 2011). To mine esophageal cancer cell specific lincRNAs, we selected the lncRNAs that were detected in four esophageal cancer cells but not in the normal-like SHEE cell and Cabili’s reference set. Under a stringent criterion (see ***Methods***), 5,400, 5,210, 4,883, 4,756, and 2,274 lncRNAs were directly predicted from SHEE, KYSE140, KYSE510, SHEEC, and TE5 cells, respectively (**Figure S2A**). Totally, 1,521 specific lincRNAs were found in esophageal cancer cells (**Table S2**).

We also predicted that lncRNAs regulated target genes and found that they are significantly enriched in cancer-related signaling pathways and extracellular matrix receptor interactions, suggesting that the interacting lincRNAs may have similar biological functions (**Figure S2B**). Furthermore, 37 potential pre-miRNAs were detected by aligning known miRNA hairpin sequences against lincRNA sequences (**Table S3**).

#### Identification of Cell-Specific Isoform Usage in Esophageal Cells

**Figure 4:**
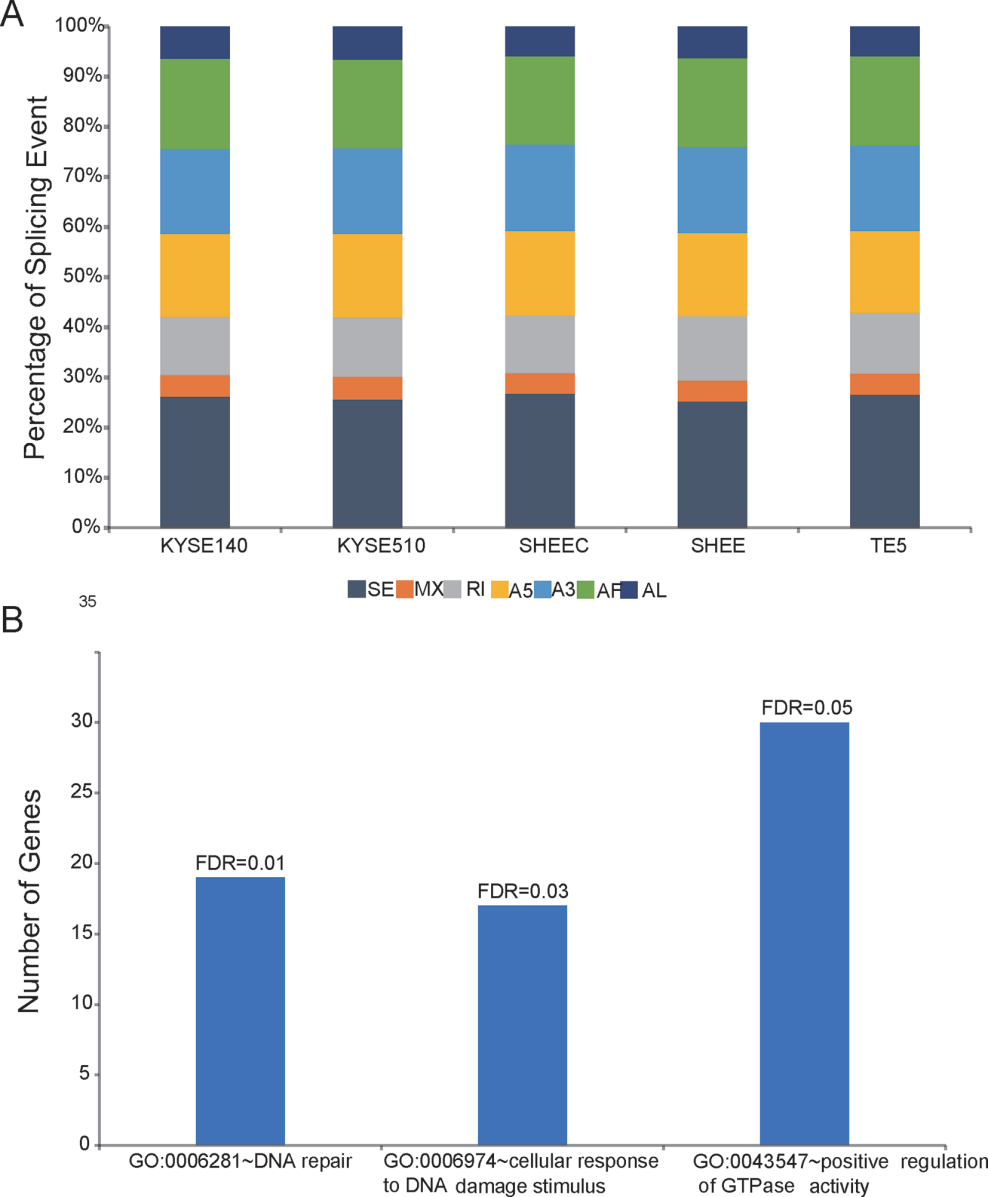
Shifted alternative splicing pattern in esophageal cells. **(A)** Percentage of splicing events in each esophageal cell line. SE, skipped exon; MXE, mutually exclusive exon; A5, alternative 5¢ splice site; A3, alternative 3¢ splice site; AF, alternative first; AL, alternative last exons; and RI, retained intron. **(B)** Differentially spliced genes between normal-like and malignant esophageal cells are significantly enriched in three Gene Ontology (GO) terms.

Employing the SUPPA package, SMRT data were also used to analyze AS events, which are classified into several categories, such as skipped exon (SE), mutually exclusive exon, alternative 5¢ splice site, alternative 3¢ splice site, alternative first, alternative last exons, and retained intron. The results showed that SE is the richest events among all AS types in all esophageal cells. This is consistent with previous findings, in which SE is the most prevalent AS mechanism in human genome (Sultan et al., 2008; Wang et al., 2008). In contrast, mutually exclusive exon only accounts for ∼5% of all AS events and is the most infrequent AS type (**Figure 4A**). Compared with four other tumor cells, AS events in the normal-like cell SHEE shows no particular preference and exclusion (÷^2 test,^ *P* > 0.05, **Figure 4A**). Score *D* is constructed to quantitatively measure the isoform usage for each gene between tumor cells and the normal-like cell. From **Figure 4B**, it can be seen that the top 500 diversely spliced genes are significantly enriched in three Gene Ontology (GO) terms “DNA repair,” “cellular response to DNA damage stimulus,” and “positive regulation of GTPase activity” (false discovery rate ≤0.05, **Figure 4B** and **Table S4**). Based on a set of differentially splicing genes identified from TCGA clinical sequencing dataset recently (Mao et al., 2019), we also verified that DNA damage and repair-related genes are significantly spliced in clinical esophageal patient samples (**Table S5**).

### Identification and Validation of Novel Fusion in Esophageal Cells

Based on searching criteria, 1,972 transcript fusions were identified from the full-length SMRT reads. The parental genes of fusion transcripts can be located from the same chromosome or from different one. However, the frequencies have no significant difference when comparing the esophageal tumor cells with the normal-like cell (÷^2 test^, *P* > 0.05, **Figure S3A**). KEGG pathway enrichment indicated that the fusion-occurring genes are in favor of biological function related with RNA processing (i.e., splicosome, ribosome, and RNA transport) and cancer signaling pathways(i.e., focal adhesion, cell cycle, and apoptosis) (**Figure S3B**).

Comparison of transcript fusion events identified by SMRT and RNA-seq assembly indicated that SMRT identifies much more fusions (**Table 2**). For example, in contrast to 39 splicing fusion found from Illumina short reads, 335 were identified from long reads in TE5 cell. ChimerDB 3.0 is an enhanced database for fusion genes from cancers (Lee et al., 2017). It archives thousands of gene fusions collected from published reports or predicted from RNA-seq transcriptome analysis. We compared the identified gene fusion pairs from PicBio SMRT data to all records in ChimerDB 3.0 (i.e., including fusions found in all cancer types). There are 2, 3, 2, 2, and 1 overlapped fusions found in TE5, SHEE, KYSE510, KYSE140, and SHEEC, respectively (**Table 2**). The fact that there were few overlaps between SMRT detection and the records in ChimerDB 3.0 suggests most fusions found from long reads are novel transcript fusions.

**Table 2:** Comparison of transcript fusions detected from PacBio data with those from Illumina reads or ChimerDB.

	TE5	SHEE	KYSE510	KYSE140	SHEEC
PacBio unique fusion	335	553	415	377	292
Illumina unique fusion	39	59	31	61	49
Found in both Illumina and PacBio	UQCC1–C22orf39 ARHGAP21—SVIL KIF13A–TPMT	HNRNPUL2–C11orf49 ARHGAP42–CNTN5 INCENP–GUCY2EP CTPS1–KCNQ4 SPAG9–LINC02071 ASAP1–KB-1568E2.1 KANSL1–ARL17B	IKBKAP–MRRF MRRF–IKBKAP GLI3–MRPL48	TNRC6B–PRR5 CERK–LINC02036 MB21D2–ATP13A4 HCG18–ERP29	–
Found in both ChimerDB and PacBio	KIF13A–TPMT NBPF1–NBPF15	KANSL1–ARL17B NBPF1–NBPF15 SLC16A3–FTH1	NBPF1–NBPF15 CDH12–HSPD1	CPSF6–C9orf3 CDH12–HSPD1	PDE4D–

**Figure 5:**
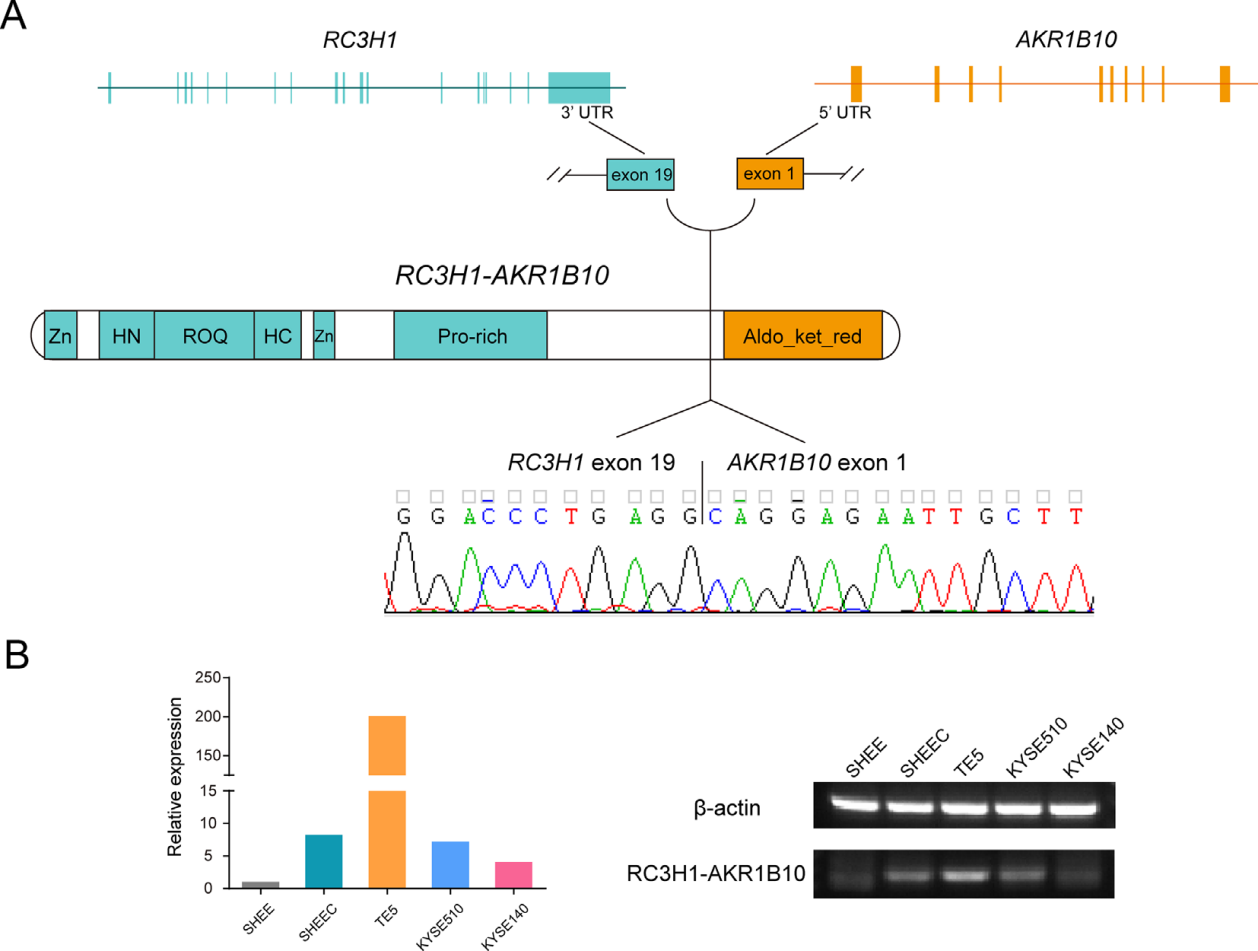
Ring finger and CCCH-type domains 1–aldo-keto reductase family 1 member B10 (RC3H1-AKR1B10) is a differentially expressed transcript fusion in esophageal cells. **(A)** Schematic of RC3H1-AKR1B10 chimeric RNA in esophageal cells. Fusion transcripts are predicted to retain intact functional regions from both parental genes. Zn: zinc finger; ROQ: Roquin domain; HN and HC: N- and C-terminal nucleotide-binding sites of Roquin domain; Aldo_ket_red: aldo/kept reductase domain. **(B)** Representative RT-PCR reactions demonstrating the differentially expressed fusion in 5 esophageal cell lines.

With the help of RNA-seq short reads, we further employed a reads filter to select six candidate fusion transcripts, which expressed comparably with their parental genes (i.e., no less than twofold of their parental genes). After manual inspection, one fusion transcript was discarded due to ambiguous sequence mapping. We evaluated the prevalence of the remaining transcript fusions in esophageal cells by real-time PCR followed by Sanger sequencing (**Table S6**). Two novel transcript fusions, ring finger and CCCH-type domains 1–aldo-keto reductase family 1 member B10 (RC3H1-AKR1B10) and NEK9-TTC21B, could be verified (**Figure 5A** and **Figure S4**). We focused on RC3H1-AKR1B10 since this transcript fusion is differentially expressed in esophageal cells, with the lowest expression in normal-like SHEE cell (**Figure 5B**). The RC3H1 protein consists of a Roquin domain, which is required for constitutive decay element-dependent RNA binding. At both N- and C-terminal sides of Roquin domain, there are regions used for nucleotide-binding. RC3H1 also contains two zinc finger motifs (Schuetz et al., 2014). AKR1B10 encodes aldo/keto reductase, which can efficiently reduce aliphatic and aromatic aldehydes (Gallego et al., 2007). The last 3¢ untranslated region exon of RC3H1 fused with the first 5¢ untranslated region exon of AKR1B10; thus, the fused protein is expected to retain intact functional regions from both of two parental genes (**Figure 5A**). Blast with default parameters was used to align the two fusion sequences to several public available RNA-seq datasets from ESCC patients. We did not find positive results from these clinical samples (data not shown).

## Discussion

Esophageal cancer is a common and highly heterogeneous malignancy. Thus, uncovering the transcriptome-wide complexity and heterogeneity will provide clues for target therapies. The median length of human gene transcripts is ∼2,500 bp; however, RNA-seq reads are only about 100–200 bp long. This indicates that novel isoforms or genes cannot be reliably inferred from short reads directly. Long reads sequencing can directly capture thousands of base pairs from single molecules, thus greatly expanding the sequencing capability. However, it is usually with lower accuracy comparing with short reads sequencing. In this study, we adopted a hybrid strategy, which combines RNA-seq and SMRT long reads sequencing, to investigate the transcriptional landscape in well-characterized ESCC cells.

Compared with the high-quality Ensembl 38 annotation of the human transcriptome, ∼15% of the spliced mappings in our results are from known gene transcripts, ∼70% are from novel isoforms of known genes, and the remaining ∼15% transcripts may represent novel isoforms of novel genes. These results suggest that isoform and gene identification are likely far from complete in esophageal squamous cells.

LincRNAs have been shown to play important roles in diverse cellular processes such as regulating key cellular pathways and recruiting the chromatin-modifying complex to specific genomic loci (Rinn and Chang, 2012). Previously, we and others have delineated several lincRNAs which are critical determinants in ESCC tumorigenesis and development (Shen et al., 2016; Lin et al., 2018; Zhang et al., 2018). Among the novel full-length transcripts, we have identified a set of esophageal cancer specific lincRNAs and pre-miRNAs potentially transcribed from them (**Tables S2** and **S3**). Furthermore, coexpressed coding genes are also found. KEGG enrichment analysis indicated that these lincRNA-regulated mRNAs are involved in a variety of cancer signaling pathways, which suggested that these cancer cell specific lincRNAs, together with its interacting partners, may be actively involved in cells transformation.

During the onset of carcinogenesis, shifted splicing of DNA repair genes has previously been documented in several cancer studies, such as BRCA1 and FANCM in breast cancer and ERCC1 in ovarian cancer (Sun et al., 2009; Sevcik et al., 2013; Peterlongo et al., 2015). However, whether or not this mechanism is active in esophageal cancer cells is largely unknown. Based on SMRT data, we found that isoform usage of DNA damage response related genes is significantly different between esophageal tumor cells and the normal-like cell (**Table S4**). Importantly, this feature can be recaptured from clinical esophageal samples (**Table S5**). In addition, we also provided evidence that activators of GTPase activity are differentially spliced between tumor and normal cells (**Figure 4B**). Although the altered splicing pattern of this group of genes is not clear in clinical samples currently, several investigations have confirmed that a few regulators of GTPase use different variants in cancers. For example, Wang et al. found that the splicing pattern of fibroblast growth factor receptor substrate 3, tuberous sclerosis 2, and RAS guanyl releasing protein 2 has significant race-related differences among prostate cancer patients (Wang et al., 2017). Furthermore, a spliced variant of ARF6 guanine nucleotide exchange factor was found to regulate the cancer cell migration and invasion (Ratcliffe et al., 2018). Intriguingly, fibroblast growth factor receptor substrate 3, tuberous sclerosis 2, and several ARF GEF and GTPase-activating protein family members are all listed at the top of spliced genes in this study (**Table S5**). Collectively, our results suggested that shift AS of genes associated with DNA damage response and GTPase signaling may contribute to ESCC pathogenesis and should be exploited for detailed mechanism in the future.

Oncogenic fusions have been found in many cancers. Growing interests have linked transcript fusion to diverse clinical applications ranging from tumor subclassification, early diagnostics, to development of effective treatment targeted this lesion (Shaw et al., 2013; Mertens et al., 2015; Yoshihara et al., 2015). However, until now, little is known for their role in ESCCs. In this study, we cataloged many novel transcript fusions and expanded the gene fusion repository in esophageal cells. We detected 1,972 novel transcript fusions from SMRT sequencing data, which are much more than those archived in cancer fusion database or fusions predicted from RNA-seq short reads. Based on RT-PCR and Sanger sequencing, we verified two novel fusion transcripts. Interestingly, our analyses uncovered a new fusion transcript between the genes RC3H1 and AKR1B10. Previous reports indicate that RC3H1 activates the deadenylation and degradation in constitutive decay elements containing mRNAs (Leppek et al., 2013; Schuetz et al., 2014). AKR1B10 is nicotinamide adenine dinucleotide phosphate-dependent aldo-keto reductase. It is highly expressed in several human cancer types such as hepatocarcinoma, nonsmall cell lung cancer and breast cancer and may play an important role in carcinogenesis (Fukumoto et al., 2005; Ma et al., 2012; Cheng et al., 2018). We demonstrated that this novel fusion is highly expressed in ESCC cells compared with the normal immortalized esophageal squamous cell (**Figure 5B**). However, they did not occur in a few ESCC patients when searching these fusions from publicly available tumor samples. One possibility is that the searched ESCC patient datasets are limited, a total of 35 patients from 4 investigations. Thus, if the above two fusions are rare (i.e., <1%), they are hardly to be detected in clinical cohorts with small size. Another possibility is that the two fusions are cell specific; thus, they cannot occur in clinical samples. The functional consequence and clinical relevance need further investigation when larger ESCC cohorts are publicly available.

## Conclusions

Sequencing technology is currently rapidly evolving. Combining PacBio SMRT platform with short reads sequencing, we have defined a large number of full-length transcripts and significantly increased the gene and isoform annotation for esophageal cells. Specifically, our investigations into the AS diversity, cancer cell specific lincRNAs, and detection of novel transcript fusions enlighten current understanding of transcriptional heterogeneity and complexity during oncogenic transformation in esophageal cells.

## Data Availability Statement

The datasets supporting the conclusions of this article are available at Gene Expression Omnibus (PRJNA515570) and in the Genome Sequence Archive in the BIG Data Center of Beijing Institute of Genomics (BIG), Chinese Academy of Sciences, under accession numbers (CRA001374). 

## Author Contributions

J-ZX, L-YX, and E-ML designed the study and the interpretation of the findings. J-ZX, Y-MC, Q-QZ, and D-ZC performed the transcriptome and other computational analyses. Y-WC, XZ, Y-RW, L-DL, YC, and QY carried out library preparation and sequencing, transcript fusion confirmation, and other experiments. J-ZX, L-YX, and E-ML oversaw the project. J-ZX wrote the manuscript that was subsequently read and approved by all coauthors.

## Funding

This work was supported by grants from National Natural Science Foundation of China (grant number nos. 81472613, 81673037, and 81772532).

## Conflict of Interest

Author Y-MC was employed by Tianjin Novogene Bioinformatics Technology Co. The remaining authors declare that the research was conducted in the absence of any commercial or financial relationships that could be construed as a potential conflict of interest.
