# Supplementary material for: Long Read Single-Molecule Real-Time Sequencing Elucidates Transcriptome-Wide Heterogeneity and Complexity in Esophageal Squamous Cells
Source: Front Genet. 2019 Oct 4;10:915. doi: 10.3389/fgene.2019.00915 (PMC6787290; doi:10.3389/fgene.2019.00915)
Supplement: Supplementary file 6 [file Table_6.doc]

**Supplementary Table 6. Primers used in this study**.

| Primer | Sequence(5' to 3') |
| --- | --- |
| β-actin-F | CATGTACGTTGCTATCCAGGC |
| β-actin-R | CTCCTTAATGTCACGCACGAT |
| NEK9/TTC21B-F | AACTCCTGACCTCAGGTGATC |
| NEK9/TTC21B-R | TCATGAGTTATTTACAGGGAGATTTATTCCA |
| NUMB/SLC31A1-F | TCGGGTGATTTTTGCTTCAGCCT |
| NUMB/SLC31A1-R | ACCATCCTGGCCAGACTCGT |
| PPP4R3A/AHR-F | TTACCGTGTTGCCCAGACTAGTCT |
| PPP4R3A/AHR-R | GAAAAGAAGAGCTGAATGCAGTGGC |
| TYMS/MAFF-F | TTAGAGGGAAGCAATCTGGTTTTG |
| TYMS/MAFF-R | CGCTTGCTGTAAGTCAGGAAAT |
| RC3H1/AKR1B10-F | GGACCCTGAGGCAGGAGAAT |
| RC3H1/AKR1B10-R | CGTCCTGCTCTCTGTTGCTG |
