# Supplementary figures and images for: Long Read Single-Molecule Real-Time Sequencing Elucidates Transcriptome-Wide Heterogeneity and Complexity in Esophageal Squamous Cells
Source: Front Genet. 2019 Oct 4;10:915. doi: 10.3389/fgene.2019.00915 (PMC6787290; doi:10.3389/fgene.2019.00915)

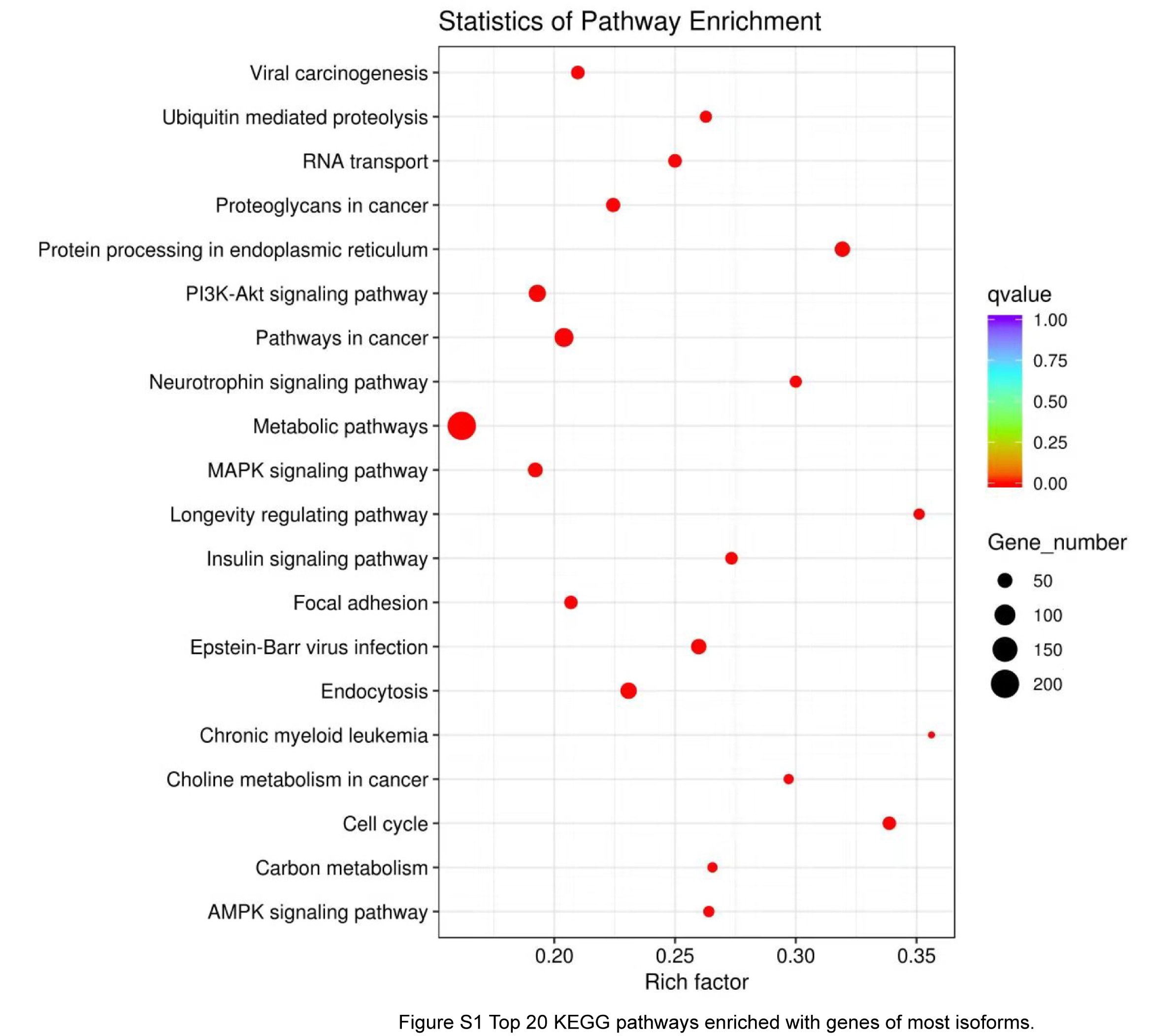

Supplement: Supplementary file 7 [file Image_1.tif]

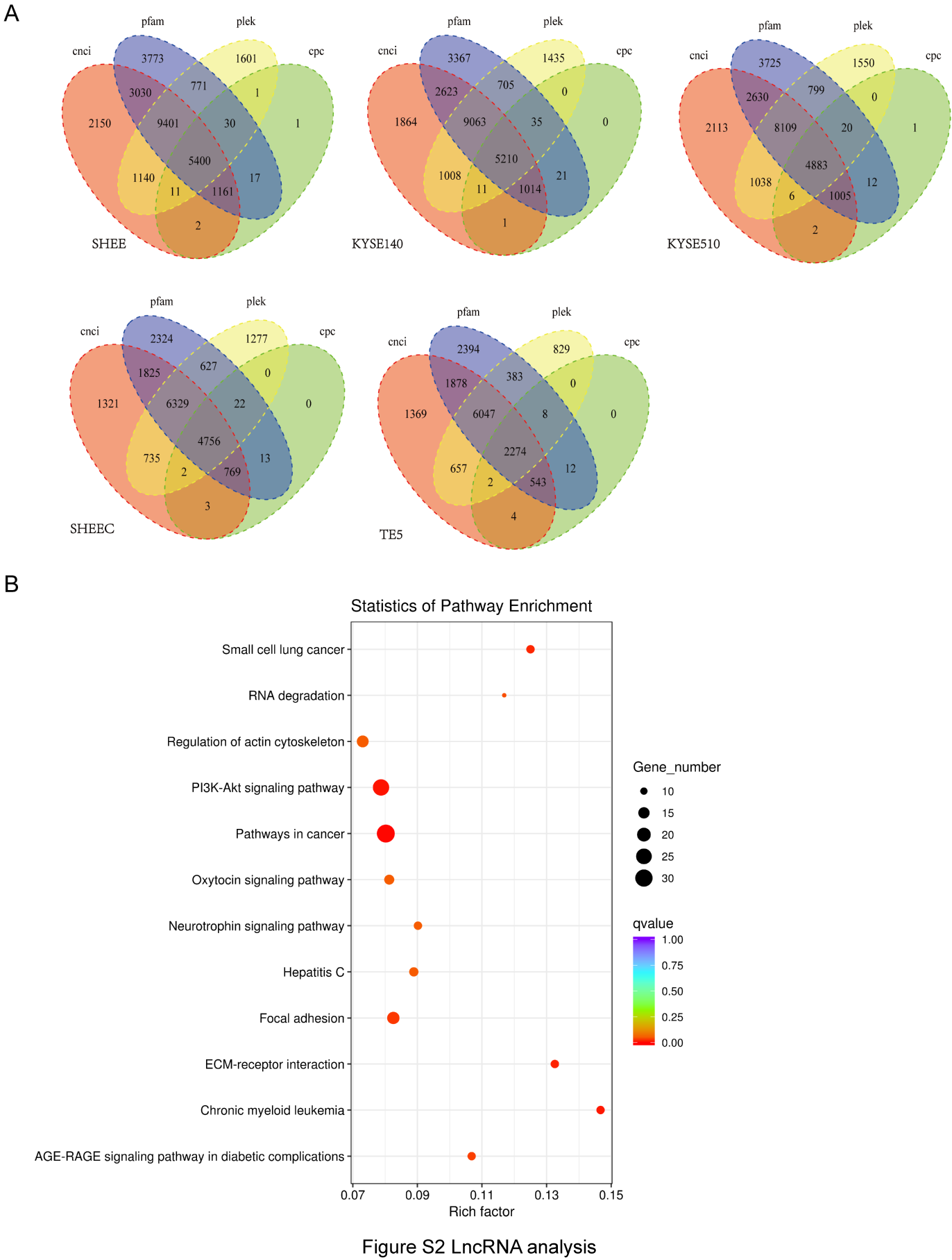

Supplement: Supplementary file 8 [file Image_2.tif]

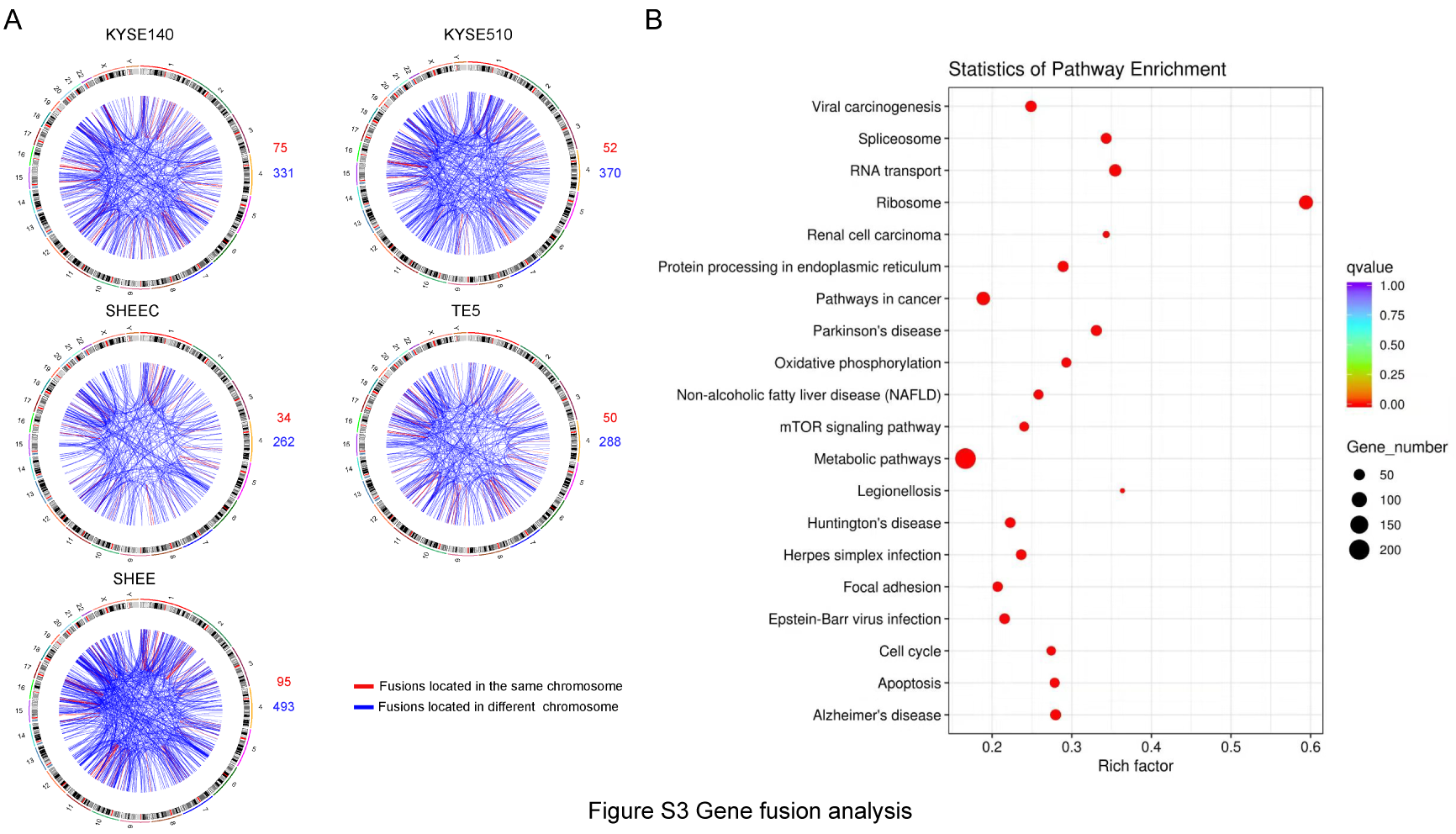

Supplement: Supplementary file 9 [file Image_3.tif]

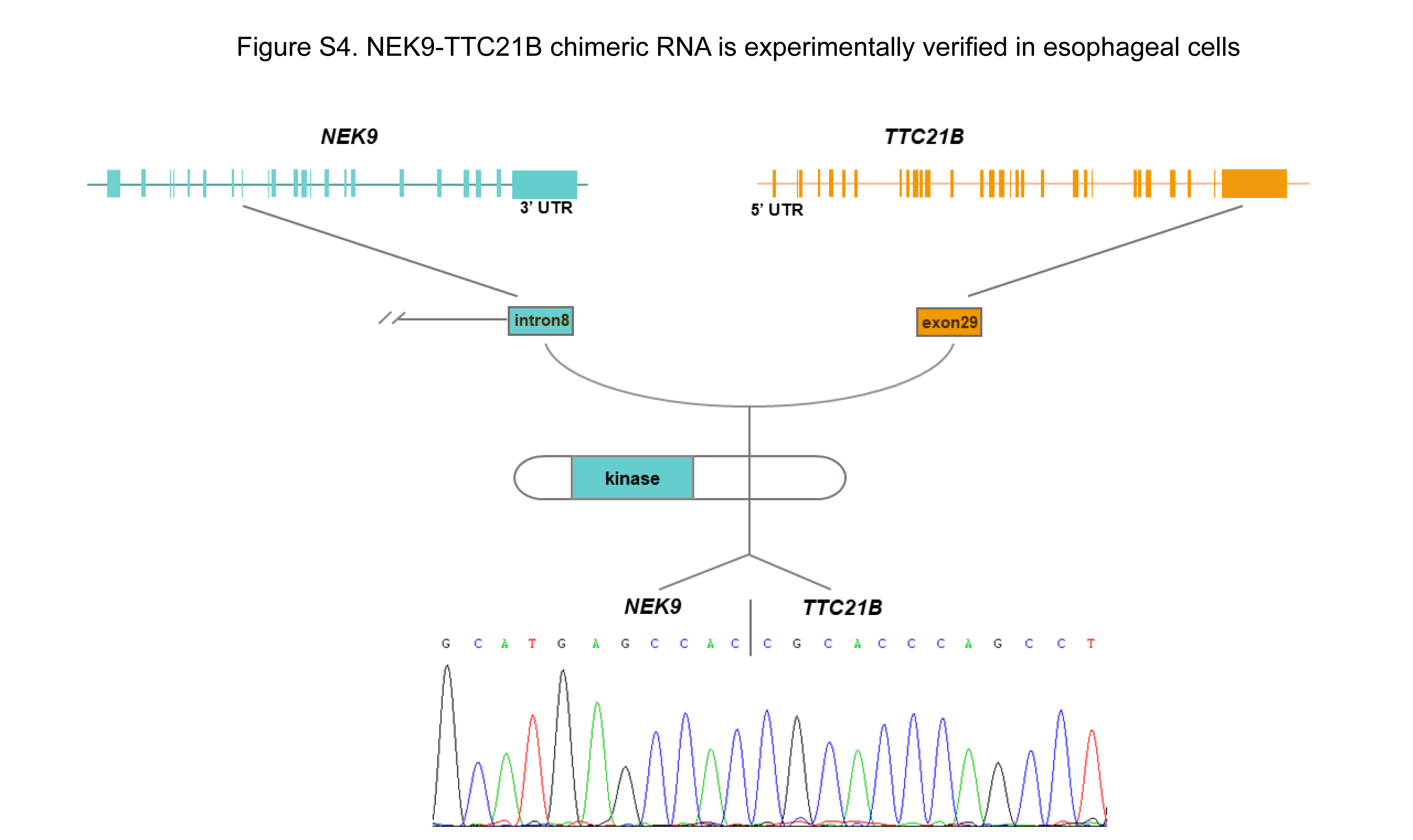

Supplement: Supplementary file 10 [file Image_4.tif]
